# Copper Complexes: Main Mechanisms as Anticancer Agents

**DOI:** 10.3390/molecules31050874

**Published:** 2026-03-06

**Authors:** Adriana Corina Hangan, Luminița Simona Oprean, Lucia Maria Procopciuc, Lucia Dican, Sidonia Gog-Bogdan, Roxana Liana Lucaciu

**Affiliations:** 1 Department of Inorganic Chemistry, Faculty of Pharmacy, “Iuliu Hațieganu” University of Medicine and Pharmacy, 400012 Cluj-Napoca, Romania; adriana.hangan@umfcluj.ro (A.C.H.); loprean@umfcluj.ro (L.S.O.); 2 Medical Biochemistry, Department of Molecular Sciences, Faculty of Medicine, “Iuliu Hațieganu” University of Medicine and Pharmacy, 400349 Cluj-Napoca, Romania; lucia.dican@umfcluj.ro; 3 Department of Surgery and ATI, Faculty of Veterinary Medicine, University of Agricultural Sciences and Veterinary Medicine, 400372 Cluj-Napoca, Romania; sidonia.bogdan@usamvcluj.ro; 4 Department of Pharmaceutical Biochemistry and Clinical Laboratory, Faculty of Pharmacy, “Iuliu Hațieganu” University of Medicine and Pharmacy, 400349 Cluj-Napoca, Romania; liana.lucaciu@umfcluj.ro

**Keywords:** copper complexes, medicinal inorganic chemistry, DNA cleavage, topoisomerases inhibitors, proteasome inhibitors, cuproptosis

## Abstract

Copper is an essential element for living organisms, being a cofactor for numerous enzymes or proteins involved in oxidation-reduction reactions, intervening in numerous metabolic processes. In recent decades, complex copper combinations have consolidated their position in medicinal chemistry, which is manifested by the increasing number of compounds that have demonstrated their efficacy following in vitro or in vivo testing. While attempting to mimic the DNA-metal complex interactions typical of cisplatin, most studies of the mechanisms of action of copper complexes continue to consider DNA as the main biological target. Starting from this, studies are focused on understanding in detail how copper complexes manage to destroy tumor cells, and this has led to the discovery of a wide range of such mechanisms of antitumor action. In this review we present the main mechanisms of action of copper complexes discovered in recent decades, from the most well-known (production of ROS following the reaction with DNA) to the newest (cuproptosis). Research into understanding the mechanisms of action of copper complexes continues to be a topic of great interest in developing new potential antitumor agents.

## 1. Introduction

Despite the successful development and clinical use of many antitumor agents, cancer remains the second leading cause of death worldwide, after cardiovascular diseases. The advent of targeted therapies has revolutionized the treatment of tumors dependent on certain oncogenes, but, for many types of cancer, progression-free survival of patients not treated with chemotherapy is relatively low, which justifies a renewed interest in cytotoxic agents including the category of complex combinations with various metal ions. The extensive clinical uses of cisplatin in the treatment of various types of tumors have placed medicinal inorganic chemistry at the frontline of the fight against cancer. Although cisplatin shows high effectiveness in the treatment of a variety of cancers, the appearance of serious side effects and chemoresistance limits the therapy. These complications have promoted novel discoveries in drug design based on different metals to reduce toxicity and open the range of cancers to be treated with some compounds [1,2,3,4,5]. Anticancer complexes containing metal ions are regarded as a highly promising category of coordination complexes for cancer treatment because of their exceptional chemical and biochemical features and mechanism of action. These complexes, also known as metallo-therapeutics, are specifically engineered to interact with biological targets such as DNA or proteins and trigger apoptosis in cancer cells while preserving the viability of healthy cells. According to studies, they can interact with cellular components in cancer cells, leading to disruption of the cell’s vital activities [6,7].

Copper is an essential element for organisms, being a cofactor for many enzymes, intervening in iron metabolism, hematopoiesis, porphyrin synthesis and in numerous metabolic processes. It is also an essential cofactor for many proteins involved in redox reactions, binding or activating oxygen. Due to its ability to oscillate between oxidized and reduced states in the biological environment, copper acts as a cofactor for enzymes involved in energy metabolism.

Unlike normal cells, tumor cells have a reduced vascularization resulting in low oxygen levels, which explains invasion, metastasis and a metabolic shift towards an anaerobic process known as the Warburg effect [8]. As a result, tumor hypoxia can be leveraged to design novel prodrugs that are selectively activated within the reducing environment of cancer cells. In this regard, copper as a metal ion becomes very attractive, since it can exist in two different oxidation states in cells. The presence of hypoxia in cancer cells promotes the reduction of Cu(II) to Cu(I), which is less possible in normal cells and thus provides a therapeutic opportunity for copper compounds to act targeted at tumors [9]. The Cu(I) ion, once formed, can catalyze the formation of ROS and RNS to induce pro-apoptotic oxidative stress.

Multiple reports show significantly increased copper levels in the serum and tumor tissues of cancer patients, supporting a role for copper and copper-binding proteins in tumor progression, angiogenesis, and metastasis. The demonstrated link between copper-related proteins and cancer development and progression has driven interest in copper coordination compounds as potential anticancer agents [10,11,12].

Over the past decades, copper complexes have established a prominent role in medicinal chemistry, as reflected by the growing number of compounds that have demonstrated efficacy in vitro and in vivo testing, with some advancing to clinical testing phase. In an effort to mimic the DNA–metal interactions characteristic of cisplatin, most mechanistic studies of copper complexes have traditionally focused on DNA as the primary biological target. Although the induction of DNA damage represents a major mechanism of action for these compounds, this antiproliferative effect does not always ensure selective cytotoxicity toward cancer cells. Consequently, research has increasingly shifted toward identifying alternative cellular targets and mechanisms of action for newly developed copper complexes [13,14,15,16,17,18].

**Figure 1:**
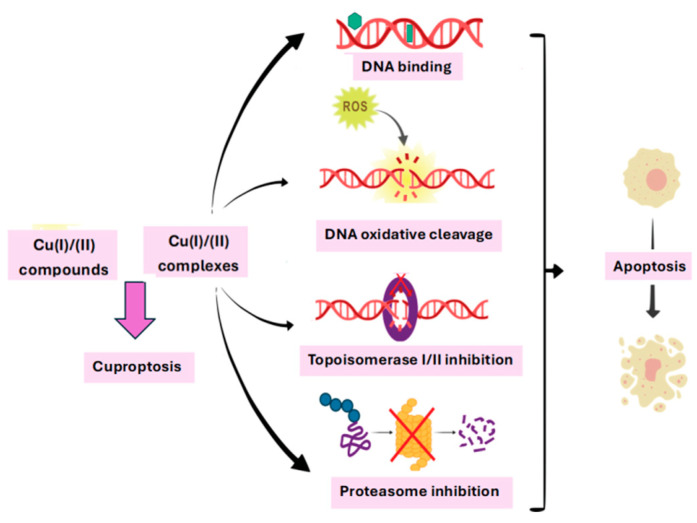
The main mechanisms of action proposed for the copper complexes as antitumor agents (adapted from [4]).

In the current review we presented the main mechanisms of action proposed for the copper complexes as antitumor agents (Figure 1).

(1) Copper complexes usually interact with DNA in a non-covalent manner. These interactions include intercalative, electrostatic, and groove binding [19,20].
(2) Once the interaction of copper complexes (Cu(I) or Cu(II)) with the DNA molecule has been achieved, there are three mechanisms involved in its destruction: an oxidative one that involves the production of ROS or RNS, a hydrolytic one that involves the formation of covalent bonds between the Cu(II) ion with the phosphate group of the nucleotides in the DNA structure and the breaking of the phosphodiester bonds at its level and photo-induced DNA cleavage [21,22,23].
(3) Inhibition of the enzyme topoisomerase I or II (role in DNA replication and transcription) or the protein disulfide isomerase is another mechanism by which copper complexes can exert their antitumor activity [24].
(4) Proteasome is another copper target that is potentially useful in cancer therapy. Copper complexes that act as proteasome inhibitors are responsible for the degradation of damaged or misfolded proteins from the cytosol and endoplasmic reticulum [25,26].
(5) In 2022, Tsvetkov et al. defined the concept of cuproptosis, representing a major advance in the understanding of copper-induced cell death. Cuproptosis is characterized by the binding of copper to lipoylated enzymes of the tricarboxylic acid cycle, leading to protein aggregation, proteotoxic stress, and ultimately cell death [27,28,29].

## 2. Interactions Between Copper Complexes and DNA

Investigations of DNA interactions with Cu(II) reveal that, similar to other divalent metal ions, Cu(II) can modulate the topology of the DNA double helix, causing unwinding or rewinding depending on the experimental conditions [30,31,32,33]. Cu(II) ions exhibit strong coordinative binding to DNA, potentially causing mutations and transcriptional errors. Their interaction can trigger conformational changes, such as the transition of poly d(GC)_2_ sequences from B-form to Z-form, which may interfere with enzyme recognition and polymerase function [34]. Moreover, Cu(II), being redox-active within a biologically relevant range, can induce oxidative DNA damage and strand breaks [35]. Cu(II) displays versatile coordination chemistry, forming numerous complexes with N-, O-, and S-donor ligands. When these ligands incorporate additional functional groups capable of hydrogen bonding, electrostatic, hydrophobic, or π–π stacking interactions, they can enhance binding affinity and confer selectivity toward specific DNA sequences, conformations, or higher-order structures. While Cu(II) ions coordinate directly to phosphate groups and nucleobases, particularly GC base pairs, Cu(II) complexes can also interact with DNA through non-covalent interactions dictated by the coordination geometry and the nature of the ligand [32,33,34].

Several studies have shown that copper complexes can cleave or intercalate DNA, acting as artificial metallonucleases [36,37,38,39]. These complexes are capable of damaging nucleic acids, leading to loss of genetic integrity or excessive activation of DNA repair pathways, ultimately triggering apoptosis and exerting anticancer effects [40,41,42,43]. In addition to covalent binding, copper complexes have been shown to interact with the DNA double helix through non-covalent mechanisms. These interactions include intercalation, electrostatic association, and groove binding along the major or minor grooves of DNA. In most cases, the copper ion functions as an inorganic modulator of the organic complex backbone, while the ligands provide DNA affinity and sequence specificity [43].

Several studies have shown that the binding mode of copper complexes to DNA is influenced by specific physicochemical properties, including ligand planarity, size, chemical nature, and coordination geometry. Generally, planar and unsubstituted ligands favor intercalative binding [40,41], whereas non-planar aromatic heterocyclic ligands exhibit reduced intercalation ability. Interactions between DNA and copper complexes can also induce structural distortions in the double helix, ultimately leading to DNA cleavage [43,44].

**Intercalation** involves the insertion of a metal complex between DNA base pairs, which distorts the double helix and can lead to mutations, chromosomal abnormalities, or double-strand breaks [7,44].

During intercalation, planar, often aromatic ligands insert between stacked DNA base pairs, with base-stacking interactions providing the principal stabilization. A key structural requirement is that the intercalating group participates in base-stacking interactions, which are a major stabilizing force for the DNA helix [38,40]. The nature of “π-stacking” is complex and influenced by factors such as electrostatic substituents and solvent environment but generally requires a planar ligand with an extensive delocalized region. For intercalation to be favorable, the energetic gain from stacking must exceed the reorganization energy required to accommodate the ligand. Intercalated groups align roughly parallel to the base pair planes, with minimal disruption to base pairing [42,43]. The primary structural effect is an extension of approximately 3.6 Å per intercalated ligand, corresponding to the normal interplanar distance for π-stacking. To accommodate this insertion, the DNA helix locally unwinds, reducing the helical twist at the intercalation site. Additional smaller geometric adjustments can reduce conformational flexibility in adjacent regions, which may prevent further intercalation at neighboring sites [19,45].

- **Groove binding**

In groove binding, a metal complex interacts with the grooves of the DNA double helix, which can increase rigidity and reduce flexibility, potentially affecting DNA replication and transcription. While most small molecules preferentially bind to the minor groove, larger molecules typically associate with the major groove [46]. Many minor groove binders target A–T rich sequences, and some small-molecule groove binders can also inhibit transcription factor activity [47,48].

Groove binding is a reversible interaction in which complexes, often exhibiting crescent-shaped topologies, complement the contours of DNA grooves, including both the major and minor grooves. The grooves differ considerably in size, shape, and chemical properties, enabling site-selective binding to A–T- or G–C-rich regions, with additional stabilization provided by hydrogen bonding and hydrophobic interactions. Because groove binding relies primarily on intermolecular forces such as electrostatic and van der Waals interactions, only minor distortions of the DNA helix are typically observed during binding [49].

- **Electrostatic interaction**

**Figure 2:**
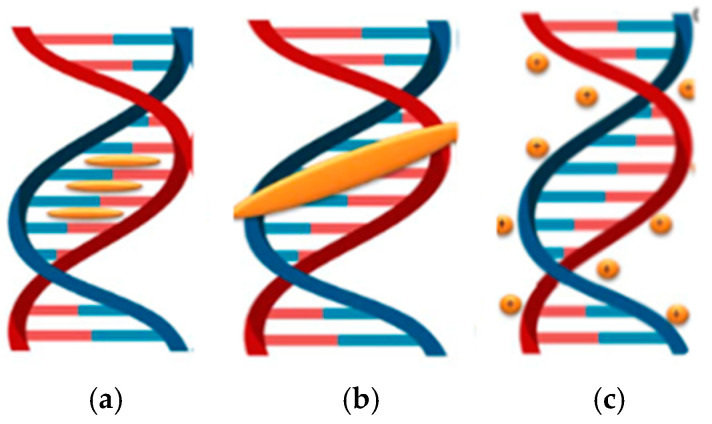
Copper complexes–DNA interactions. (**a**) intercalation; (**b**) groove binding; (**c**) electrostatic interaction.

Electrostatic binding is a non-covalent interaction in which most cationic transition-metal-based drug complexes are associated with the negatively charged oxygen atoms of the DNA phosphate backbone [19,20]. By neutralizing this negative charge, repulsive forces between adjacent phosphate groups are reduced, stabilizing the double helix. As this binding occurs externally along the sugar-phosphate backbone, it induces notable stabilization of the DNA helix, causes conformational changes, and can ultimately disrupt the secondary structure and inhibit DNA replication. Although the strength of electrostatic interactions is generally lower than that of other binding modes, such as intercalation, they often act synergistically with other surface non-covalent interactions to enhance overall DNA-binding affinity at the binding site [49,50,51]. In Figure 2 the primary modes of interaction between Cu(I)/Cu(II) complexes and DNA are illustrated.

Cu(II) complexes with amino acids and peptides predominantly interact with DNA via the minor groove. The coordinating side chain of histidine, and its position within tripeptides (central or C-terminal), influences whether the non-covalent peptide–DNA interaction is further reinforced by covalent binding of the metal center to nucleobases or the phosphate backbone. Non-coordinating amino acid side chains can modulate binding through additional hydrogen bonding, hydrophobic interactions, steric effects, or, in the case of aromatic residues, intercalation. These observations are mainly inferred from binding affinity data, with molecular modeling providing additional support [52,53,54].

The tetrahedral bis(phenanthroline)copper(I) complex binds preferentially or exclusively in the DNA minor groove [55,56]. There has been some debate as to whether the non-covalent binding at this level is associated or not with a partial intercalation of the complex between base pairs. Some research groups suggested a binding by partial intercalation of one phenanthroline ring between a T-3′,5′-A step, with the other phenanthroline ring lying within the minor groove approximately parallel to the helix axis [57,58]. Other models suggested a DNA minor groove binding of the complex without intercalation phenomena [59]. The mono(phenanthroline)copper(I) complex, [Cu(phen)]^+^, also binds to DNA by intercalation while DNA binding of free phenanthroline may be cooperative and induced only by prior binding of [Cu(phen)_2_]^+^ [57]. For complex [Cu(phen)_2_]^2+^ Sigman [60] suggests an initial reduction to [Cu(phen)_2_]^+^ induce by the presence of a thiol reducing agent. Taking into consideration that the complex [Cu(phen)_2_]^+^ is more active than [Cu(phen)]^+^ Meunier et al. have prepared 2- and 3-clip-phen derivatives containing two phenanthroline ligands linked by an alkoxo-bridge in order to favor the coordination of two phenanthroline units around the same copper ion. Copper(II) complexes with “clip-phen” derivatives exhibit oxidative nuclease activity that is 2 to 60 times higher than that of copper(II) complexes with 1,10-phenantroline [61]. In order to improve the modest sequence selectivity the clip-phen strategy was used to prepare a number of 3-clip-phen conjugated with DNA binders (acridine, distamycine, spermine, etc.) leading to encouraging results [62,63,64]. Simulation made using a combination of theoretical methods revealed that several factors such as the planarity of the ligand, a better interaction with the DNA and the existence of interactions with the minor-groove contribute to the enhanced efficiency of the copper(II) complexes with clip-phen ligands as compared to other structurally similar complexes [65].

Cu(II) complexes with phenanthroline-like ligands with an extended planar p-system like dipyrido[3,2-d:2′,3′-f]quinoxaline (dpq) and dipyridophenazine (dppz) bind more strongly to DNA than [Cu(phen)_2_]^+^ and [Cu(phen)]^+^ [66,67,68,69]. Effective intercalation can facilitate the DNA cleavage process by deforming the DNA structure. Due to the lack of an extended planar aromatic system, [Cu(bpy)_2_]^+^ does not intercalate into DNA but interacts electrostatically with the negatively charged sugar-phosphate backbone [70]. The size of the aromatic system also affects the groove preference. In contrast to [Cu(phen)_2_]^+^ and [Cu(dpq)]^+^, [Cu(dppz)]^+^ interacts with DNA via the major groove. As expected, the extended p-system also leads to a higher binding affinity compared to [Cu(phen)_3_]^2+^.

Porphyrins, with their planar tetrapyrrole framework, are ideally suited to stack on G4 quartets and are well-known as quadruplex DNA binders. Intercalation has also been proposed, particularly in regions with extended guanine stretches [71]. While Cu(II) complexes with porphyrins or related ligands are extensively studied as quadruplex binders, they can also interact with conventional DNA in a manner similar to their metal-free (cationic) counterparts. The porphyrin core promotes both intercalation into DNA and end-stacking on the G-tetrad of quadruplex DNA, although steric factors strongly influence binding. In general, Cu(II) porphyrin complexes carry a positive charge.

Cu(II) complexes with N,N′-bis-salicylidene-ethylenediamine (salen) and N,N′-bis-salicylidene-1,2-phenylenediamine (salphen) exhibit strong interactions with DNA, with binding affinity often enhanced by positively charged substituents [72,73]. Depending on the Cu(II) coordination geometry and ligand planarity, both intercalation and groove binding can occur. For instance, [Cu(salen-NMe_3_)]^2+^ binds primarily via the groove, whereas the analogous [Cu(salphen-N(CH_3_)_3_)]^2+^ and [Cu(salnaph-N(CH_3_)_3_)]^2+^ interact through intercalation [73,74]. Despite evidence that various Cu–thiosemicarbazone and Cu–semicarbazone complexes appear to interact with DNA, systematic studies correlating structure and binding affinity are lacking, and current data are insufficient to establish definitive structure–binding mode relationships.

Cu(II) Schiff base complexes featuring N,N′-bis-5-(triethylammoniummethyl)-salicylidene-2,3-naphthalenediiminato as the ligand interact with DNA via intercalation of the naphthalene moiety into the base stack, exhibiting selective binding for AT over GC sequences [51].

Table 1 provides a summary of investigations on the interactions of selected Cu(II) complexes with DNA.

## 3. DNA Denaturation Induced by Copper Complexes

Generally, Cu(II) complexes can cleave DNA through three main mechanisms: (1) oxidative cleavage, in which the complexes generate reactive oxygen species (ROS) such as singlet oxygen (^1^O_2_), superoxide radical (O_2_^•−^), and hydroxyl radical (HO•), etc., leading to DNA strand breakage [87,88,89,90]; (2) hydrolytic cleavage, in which Cu(II) complexes catalyze the hydrolysis of phosphodiester bonds in the DNA backbone, resulting in DNA cleavage [91,92,93]; and (3) photoinduced cleavage, in which irradiation of copper complexes produces ROS that cause DNA strand breaks [94,95].

### 3.1. Oxydative Cleavage

ROS generated by Cu(II) complexes play a crucial role in DNA cleavage. Upon approaching DNA, Cu(II) complexes can readily react with glutathione (GSH), a strong cellular reductant, resulting in the formation of Cu(I) complexes. Many Cu(II) complexes possess high oxidation potentials, whereas GSH has a low reduction potential [88,89,90]. Electron transfer from GSH to the Cu(II) complexes occurs efficiently, forming Cu(I) species; the larger the potential difference between the complex and GSH, the faster the electron transfer. These rapidly moving electrons can subsequently react with surrounding O_2_ or H_2_O molecules to generate highly reactive ROS, such as superoxide (O_2_^•−^), singlet oxygen (^1^O_2_), and hydroxyl radicals (HO•). Experimental studies further indicate that fast electron movement can induce radical ions on the complexes, contributing additionally to HO• generation [96,97]. Cu(II) complexes with polypyridyl ligands, including 1,10-phenanthroline, feature conjugated *π*-bonds. These *π*-systems can delocalize electrons through the central Cu(II) atom, allowing the *π*-electrons to move across the entire complex, which often results in strong DNA-binding affinity [21,22]. The oxidative mechanism begins with the reduction of the metal center, which reacts with molecular oxygen to generate superoxide radicals (O_2_^•−^). SOD then catalyzes the dismutation of O_2_^•−^ into O_2_ and hydrogen peroxide (H_2_O_2_), the latter being less reactive but highly diffusible. The metal ion subsequently reacts with H_2_O_2_ to produce highly reactive hydroxyl radicals (HO•), which can damage biological molecules such as nucleic acids, lipid membranes, and proteins via Fenton- or Haber–Weiss-type reactions. This mechanism constitutes a one-electron reduction cascade of molecular oxygen, leading to ROS generation and initiating DNA strand scission. The majority of Cu(II) complexes cleave DNA through oxidative pathways, with numerous studies focusing on Cu(II) complexes bearing N-substituted sulfonamide ligands [96,97,98,99,100,101].

**Figure 3:**
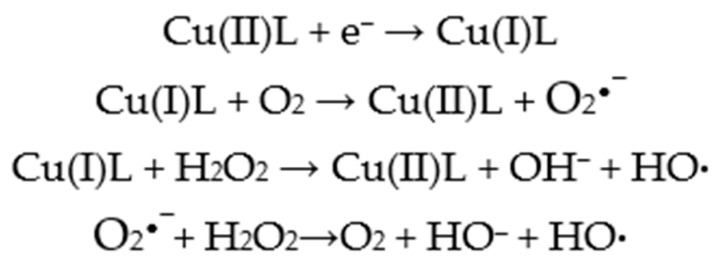
ROS generation mediated by redox-active Cu(I)/Cu(II) ions.

Figure 3 illustrates the generation of ROS by copper complexes via Fenton or Haber-Weiss reactions.

### 3.2. Hydrolytic Cleavage

This mechanism of DNA damage occurs when the mono or dinuclear Cu(II) complexes form covalent interactions with the phosphate groups of nucleotides within the DNA structure, leading to cleavage of the phosphodiester bonds and subsequent degradation of the nucleic acid molecule. These Cu(II) complexes act as “chemical nucleases” through several pathways, namely: nucleobase oxidation, phosphate ester hydrolysis, and deoxyribose oxidation. The Cu(II)-TACN complex (TACN = 1,4,7-triazacyclononane) Sexhibits a predominant cleavage hydrolysis mechanism [102], while other Cu(II) complexes called “molecular scissors” can destroy the DNA molecule by cleavage in its single- or double-stranded structure [103].

**Figure 4:**
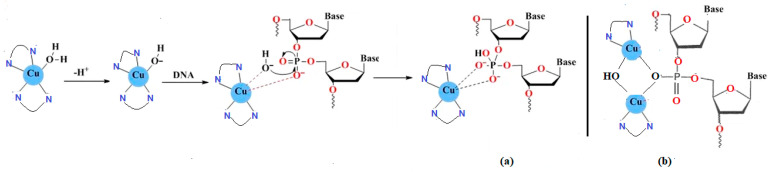
Cu(II) complexes interactions with DNA phosphate moieties. (**a**) mononuclear Cu(II) complex; (**b**) dinuclear Cu(II) complex.

This type of mechanism was observed in the case of complexes where Cu(II) ions were connected through hydroxyl bridges {Cu_2_(μ-OH)_2_^2+^}, but also in the mononuclear complexes of copper. These complexes have a high nuclease activity even in the presence of inhibitors like DMSO or sodium azide. This proves that in this case the cleavage of DNA is done independently of oxygen, through a process different from oxidative degradation. The mono- or di-nuclear Cu(II) complexes which contain labile bonds can be used as accelerators of the hydrolysis of the phosphodiester groups and can finally lead to the destruction of DNA (Figure 4) [104,105].

### 3.3. Photo-Induced DNA Cleavage

Oxidative DNA cleavage may occur either upon the activation with a reducing agent or irradiation with visible light of long wavelength (600–800 nm) or ultra-violet light (320–380 nm). The methodology based upon irradiation with visible light is particularly attractive for its potential use in photodynamic therapy (PDT) of cancer. PDT is a noninvasive treatment combining the use of red light and a photosensitizing drug that can transfer the excited state energy acquired upon photoexcitation to molecular oxygen generating singlet-oxygen ultimately responsible for oxidative cellular damage [106,107,108].

Copper induces DNA cleavage through the generation of reactive oxygen species. Likewise, light-assisted excitation of Cu(II) complexes in the red region of the electromagnetic spectrum promotes the formation of various reactive species, leading to anticancer activity via a photodynamic mechanism. Complexes that exhibit photo-induced DNA cleavage offer significant advantages over conventional “chemical nucleases”, as they do not require additional reagents such as reducing agents or H_2_O_2_ to exert their activity. Moreover, photo-activated DNA-cleaving compounds typically display localized therapeutic effects and are non-toxic in the absence of light [109].

PDT is regarded as a promising targeted treatment strategy with significant potential to reduce collateral damage associated with conventional chemotherapy. PDT is based on localized, light-induced cytotoxicity, primarily mediated through the generation of ROS. Incubation of cells with complexes, both in the presence and absence of light activation, enables evaluation of their photodynamic efficiency. It has been shown that IC_50_ values can be reduced by several hundred-fold compared to treatments without irradiation [109,110]. Although a major limitation of PDT is its dependence on oxygen in the typically hypoxic tumor microenvironment, the pronounced redox activity of Cu(II) makes it an attractive candidate for controlled cytotoxicity, as it can engage in oxygen-independent ROS-generating reactions [111].

With respect to their analogues promoting oxidative DNA cleavage in the presence of reducing agents, photo-active complexes have the significant advantage of promoting double-strand cleavage in the absence of co-reactants. In addition, compounds cleaving DNA on photo-activation are known to show localized effects in therapeutic applications and are non-toxic unless irradiated [109]. Cu(II) complexes showing photo-induced DNA cleavage facilitate strand scission through the photosensitization of singlet oxygen or the generation of other diffusible reactive oxygen intermediates, the hydroxyl radical being frequently incriminated [112,113,114,115,116]. The generation of ROS may occur as the result of the presence of a photosensitizer ligand bearing photoactive groups; in addition, the ligands linked through a Cu(II) center may involve the metal based d–d and/or charge transfer bands in the photoexcitation process resulting in the formation of ROS. The latter mechanism is responsible for ROS formation even in case of complexes with ligands that do not bear photoactive groups. Regardless of the photoexcitation mechanism, the ligands linked to the metal center or the complex as a whole have to exhibit features allowing for an efficient DNA—complex interaction [114,115,116].

Ternary Cu(II) complexes containing amino acids such as L-methionine or L-lysine and a phenanthroline base are effective DNA photocleavers. Complexes of the type [Cu(Ln)(phen)](ClO_4_) incorporating an NSO-donor Schiff base (HLn) and the N,N-donor heterocycle base, 1,10-phenanthroline (phen), exhibit pronounced cleavage of supercoiled DNA upon irradiation with red light at 700 nm. The *d–d* and CT electronic bands of these Cu(II) complexes play a critical role in DNA cleavage reactions. The underlying mechanisms depend on both the ligand type and the excitation energy. Whereas UV irradiation typically induces DNA cleavage via a type-II pathway involving singlet oxygen (^1^O_2_), red-light activation promotes DNA cleavage through multiple pathways, including type-I, type-II, and photo-redox pathways. Cu(II) complexes containing phen as a DNA-binding moiety and a thiomethyl-substituted Schiff base as a photosensitizer cleave supercoiled DNA to its nicked circular form via a type-II mechanism under red-light irradiation. The binary complex [Cu(dpq)_2_(H_2_O)](ClO_4_)_2_ (dpq = dipyridoquinoxaline) induces DNA cleavage through a photo-redox pathway upon irradiation at 694 nm. Similarly, the binuclear complex [Cu_2_(RSSR)_2_], where H_2_RSSR is a Schiff base derived from 2-(thioethyl)salicylaldimine, cleaves supercoiled DNA at 632 and 694 nm, involving sulfide (S^2−^) species in a type-I process and hydroxyl radicals (•OH) via a photo-redox mechanism [109,117].

Cynthia Al Hageh et al. investigated sterically hindered Cu(I) complexes and found that Cu(dsbtmp)^2+^ (dsbtmp = 2,9-didecylbutyl-3,4,7,8-tetramethyl-1,10-phenanthroline) exhibited in vitro phototoxicity against two cancer cell lines: A375 human malignant melanoma and A549 human lung carcinoma. Their results demonstrated that Cu(dsbtmp)^2+^ induces DNA damage under dark conditions and further enhances DNA damage through increased ROS generation upon light exposure in both melanoma and lung cancer cells [118].

Jung et al. reported a novel photosensitizer, CA9-BPS-Cu(II), which demonstrated excellent in vitro and in vivo activity against MDA-MB-231 breast cancer cells, with selective targeting of CD133-positive subpopulations. The complex integrates the redox properties of Cu(II), a boron dipyrromethene (BPS) scaffold as the photosensitizer, and an acetazolamide (CA9) moiety to target cancer stem cells via CA9 binding. In vivo xenograft tumor models showed pronounced tumor growth inhibition, particularly upon activation with 660 nm laser irradiation. Minimal antitumor effects were observed in the absence of light, highlighting the therapeutic potential of photo-activated treatments [106,119].

Table 2 presents some copper (II) complexes that act as photocleavage agents.

Despite growing evidence supporting the use of copper complexes in PDT, challenges such as increased toxicity [125] and light-induced degradation [126] persist, and clinical evaluation remains limited. Overall, copper complexes exhibit substantial promise as photosensitizers in PDT; however, critical issues such as tissue selectivity, cytotoxicity, photostability, and limited light penetration in clinical settings must be addressed. Future research should focus on optimizing molecular design, integrating multimodal therapeutic strategies, and advancing copper complexes into clinical trials to enhance their therapeutic potential in cancer treatment.

## 4. Copper Complexes as Topoisomerases and Protein Disulfide Isomerase Inhibitors

Inhibition of topoisomerase I or II, which are essential for DNA replication and transcription, and protein disulfide isomerase constitutes an additional pathway through which copper complexes exert antitumor activity [24].

### 4.1. Copper Complexes as Topoisomerases Inhibitors

The essential roles of topoisomerases make them important targets in cancer therapy. Topoisomerases (Tops) are crucial enzymes that maintain genome stability by regulating DNA topology during DNA metabolism. Cells dynamically control DNA supercoiling, a process necessary for transcription, replication, and cell division. By modulating DNA winding and unwinding, Tops play key roles in DNA replication and transcription [127,128]. Based on their catalytic mechanisms, Tops are classified into two major families: Type I and Type II.

Type I Tops relieve DNA supercoiling by cleaving and religating a single strand of the DNA double helix through either a strand-passage or controlled-rotation mechanism, thereby removing both positive (overwinding) and negative (underwinding) supercoils. In contrast, type II Tops generate transient double-stranded DNA breaks and pass an intact DNA duplex through the break before religating the strands, enabling the removal of supercoils, DNA knots, and catenated (intertwined) DNA molecules. In both cases, DNA cleavage is stabilized by the formation of a covalent intermediate between the enzyme and DNA via an active-site tyrosine residue. In humans, six Tops have been identified: Top1β, mitochondrial Top1β, Top2α, Top2β, Top3α, and Top3β [128].

Tops transiently introduce single- or double-stranded DNA breaks during their catalytic cycle, which can become permanent and result in DNA damage and cell death. Agents that interfere with Tops function are generally classified into two categories: catalytic inhibitors and topoisomerase poisons [129,130]. Top poisons stabilize the covalent Tops–DNA cleavage complex, forming a ternary Tops–drug–DNA intermediate that prevents DNA religation. This leads to the accumulation of single- and double-strand breaks and triggers apoptosis [131]. In contrast, catalytic inhibitors target other steps of the catalytic cycle, most commonly by interacting with or near the ATPase domain and inhibiting ATP hydrolysis [130,131,132,133]. The inhibitory activity of Top inhibitors is often enhanced upon complexation with Cu(I)/Cu(II) ions. Copper-based Top1, Top2, or dual Top1/2 inhibitors are predominantly mononuclear Cu(II) complexes coordinated with a variety of ligands. Several strategies have been proposed for the design and development of Top inhibitors based on the physicochemical properties of the ligands [134]. When Cu(II) complexes act as Top1 and/or Top2 inhibitors, they primarily target DNA through direct interactions such as intercalation or cleavage; their antiproliferative activity is further amplified by ROS generation and interactions with additional molecular targets [135,136].

Copper-based Top inhibitors promote DNA strand breaks either by forming ternary complexes with Tops and DNA or by acting as catalytic inhibitors through DNA intercalation at the Top binding site and the generation of reactive oxygen species [133]. As a result, copper complex–based Top inhibitors represent highly active anticancer agents with significant potential for future cancer therapy.

Both Top1 and Top2 copper-based inhibitors induce DNA damage. Top2 poisons prevent DNA religation and stabilize enzyme–DNA complexes containing double-stranded DNA breaks [135]. In contrast, Top1 poisons generate single-stranded DNA breaks and activate associated signaling pathways; collisions between Top1 cleavage complexes and DNA replication forks subsequently result in double-strand breaks [135,136,137,138]. Unlike poisons, Top2 catalytic inhibitors do not form cleavable complexes but instead inhibit enzymatic activity and induce G2 phase cell cycle arrest via a decatenation checkpoint distinct from the DNA damage checkpoint [132,133,134,135,136,137,138,139]. Many copper complexes have been shown to inhibit tops, functioning either as Top poisons and/or as catalytic inhibitors. Holder et al. reported a Cu(II)–thiosemicarbazone complex that acted as a human Top IIα poison and exhibited pronounced anti-proliferative activity against several colon cancer and aggressive breast cancer cell lines [140,141]. Desideri et al. described a copper(II)–oxindolimine complex that, at a concentration of 50 μM, completely inhibited Top Iβ–mediated DNA relaxation, acting as a catalytic inhibitor rather than a poison [142].

Afsan et al. synthesized a Schiff base copper(II) complex derived from benzenesulfonamide, which displayed selective cytotoxicity against the MCF-7 cell line. Both in silico and in vitro DNA studies indicated a non-covalent intercalative binding mode, along with efficient DNA cleavage through an oxidative mechanism. Additionally, the copper complex showed notable Top I inhibition, demonstrating a dual mechanism of DNA damage via intercalation and Top I inhibition [142]. Conner et al. investigated a series of copper(II)–benzoylpyridine thiosemicarbazone compounds for their ability to inhibit human Top IIα and their potent activity against human breast cancer cell lines (MDA-MB-231 and MCF7) [143]. Recently, Lisic et al. developed two novel thiosemicarbazone–copper(II) complexes incorporating 2-propionylthiazole ethylthiosemicarbazone and 2-propionylthiazole tert-butylthiosemicarbazone. Their findings demonstrated that both complexes effectively reduced the viability of MCF7 and MDA-MB-231 breast cancer cell lines, whereas the free ligands showed considerably lower activity [139]. Sandhaous et al. reported a Cu(II) thiosemicarbazone complex, [Cu(acetylTSC)Cl]Cl **·** 0.25C_2_H_5_OH, that induces DNA damage by inhibiting Top IIα. The complex blocked the enzyme’s ability to relax DNA, whereas the free ligand had no effect on Top activity. Additionally, it enhanced DNA cleavage, suggesting that it functions as a poison inhibitor of human Top IIα. The complex also reduced the viability of several colon cancer cell lines (HTC-116, Caco-2, and HT-29) as well as a non-tumoral colon cell line (CCD-18Co), exhibiting greater anticancer activity than the clinical drug etoposide [144].

Palanithu et al. synthesized a series of bis(thiosemicarbazone) ligands and their copper complexes. Among these, the compounds derived from glyoxal-bis(4-methyl-4-phenyl-3-thiosemicarbazone) showed the highest cytotoxicity, particularly against HCT116 colon cancer cells. Treatment of HCT116 cells with these complexes resulted in a marked increase in cellular copper levels, primarily within the cytoplasm, compared to untreated controls. Mechanistic studies indicated that the complexes inhibit DNA synthesis and interact with DNA via partial intercalation [145]. Table 3 lists several copper complexes that act as Top inhibitors.

The development and optimization of copper complexes as Top inhibitors involve structural modifications guided by strategies such as scaffold hopping, pharmacophore hybridization, bioisosteric replacement, and conformational constraints [170,171]. In general, rigidifying the ligand’s heterocyclic structure through coordination with copper imparts a planar geometry, which enhances DNA intercalation and promotes the formation of the Top-DNA ternary complex more effectively than the ligand scaffold alone.

### 4.2. Copper Complexes as Protein Disulfide Isomerase Inhibitors

Protein disulfide isomerase (PDI) belongs to the mercaptan isomerase family and is predominantly localized in the endoplasmic reticulum (ER). To date, at least 21 members of the PDI family have been identified. PDI is essential for proper protein folding, the correction of misfolded proteins, and the catalysis of disulfide bond formation, rearrangement, and breaking. Consequently, dysregulated PDI activity is associated with a wide range of diseases, including cancer, infections, immune and metabolic disorders, thrombosis, and neurodegenerative conditions. Elevated intracellular levels of PDI promote cancer cell proliferation, metastasis, and invasion, highlighting its potential as a cancer biomarker. Because cancer cells rely heavily on protein synthesis, PDI-mediated disulfide bond formation is particularly critical, resulting in higher PDI expression compared to normal cells. Inhibition of PDI can trigger ER stress and activate the Unfolded Protein Response (UPR) pathway, ultimately inducing apoptosis in cancer cells [172,173].

As a copper-binding protein, PDI has emerged as a promising cellular therapeutic target in cancer treatment. Inhibitors that target mitochondrial-resident PDI can trigger cancer cell death through non-oxidative stress mechanisms [174]. PDI exhibits a strong affinity for copper and plays a crucial role in maintaining intracellular copper redox homeostasis, which is essential for catalyzing disulfide bond formation. Elevated PDI expression has been reported in multiple cancers, including lung, kidney, brain, ovarian, and prostate cancers, highlighting its potential utility as a diagnostic biomarker [174,175,176]. Conversely, reduced PDI levels have been associated with improved patient survival, suggesting that PDI supports cancer cell survival and progression. Accordingly, studies indicate that PDI inhibitors may function as effective chemotherapeutic agents by disrupting key processes required for cancer cell maintenance and growth [175,176,177,178].

Some studies assessed intracellular levels of reduced thiols in cells treated with metal complexes, supporting the hypothesis that Cu(I) derivatives coordinated to pyrazolyl ligands or Cu(II) complexes bound to thiosemicarbazones can effectively target PDI in colon cancer cells. Such interactions disrupt cellular redox homeostasis, shifting it toward a more reduced state. Moreover, morphological analyses showed that both complexes caused a modest increase in mitochondrial size, decreased electron density in the inner membrane and matrix, and induced alterations in cristae structure [179,180]. Compounds that target and inhibit mitochondria-resident IPRs (iron regulatory proteins) can trigger cancer cell death through non-oxidative stress mechanisms. Additionally, IPRs possess copper-binding affinity and play a crucial role in controlling the intracellular redox state of copper ions, which are involved in catalyzing disulfide bond formation [179]. Carcelli et al. developed a set of square-planar Cu(II) complexes incorporating substituted salicylaldehyde thiosemicarbazone ligands. Both the free ligands and the corresponding copper complexes were tested against a diverse panel of cancer cell lines (HCT-15, LoVo, A375, BxPC3, PSN1 and HEK293), where they displayed marked cytotoxic activity. In three-dimensional spheroid models of colon cancer (HCT-15 and PSN1), the complexes showed up to 60-fold higher activity compared with cisplatin. Morphological analyses of LoVo cells further indicated that these compounds strongly suppress PDI activity, exhibiting low-micromolar IC_50_. Overall, the study highlights the potential of long-established Cu(II)–thiosemicarbazone systems to be re-engineered as anticancer agents with novel cellular targets, enhanced efficacy, and improved selectivity [180].

## 5. Copper Complexes as Proteasome Inhibitors

The proteasome represents another copper-associated target with significant potential in cancer therapy. This catalytic protein complex is responsible for degrading misfolded or unwanted intracellular proteins and regulates key cellular processes in both normal and malignant cells. Cancer cells exhibit elevated proteasome activity to sustain rapid growth, and inhibition of this system disrupts protein homeostasis and ultimately triggers apoptotic cell death [181,182,183]. 26S proteasome is a large, multicatalytic protease complex composed of a central 20S proteolytic core flanked by two 19S regulatory caps. The 20S core exhibits chymotrypsin-like, trypsin-like, and caspase-like protease activities [184]. This complex is responsible for degrading proteins that have been tagged with ubiquitin molecules. The proteasome recognizes polyubiquitin chains, allowing it to selectively degrade the ubiquitinated protein [185]. The complex mediates the degradation of damaged or misfolded proteins originating from the cytosol and the endoplasmic reticulum. Moreover, the proteasome is essential for the rapid turnover of key regulatory proteins, such as cell cycle regulators and transcription factors. Through these functions, the ubiquitin–proteasome pathway plays a central role in numerous cellular processes, including cell cycle progression and apoptosis. Notably, cancer cells exhibit greater sensitivity to proteasome inhibition than non-tumoral cells [14,186].

Bortezomib, a reversible proteasome inhibitor, was the first drug approved by the US Food and Drug Administration (FDA) for the treatment of multiple myeloma and mantle cell lymphoma. Its clinical application, however, was limited by severe toxicities, low efficacy against solid tumors, and tumor relapse in treated patients. Subsequently, the FDA approved two additional proteasome inhibitors, carfilzomib and ixazomib, though their clinical use has also been constrained due to the development of drug resistance [187,188].

Cell-free studies have demonstrated that Cu(II) ions can inhibit 20S proteasome activity by inducing conformational changes. Several novel copper-based proteasome inhibitors have since been developed, exhibiting selective inhibition of proteasomic pathways and offering potential for controlling abnormal cancer cell growth [189,190,191]. Copper complexes demonstrated potent proteasome inhibition and apoptosis-inducing activity in tumor cells, while exerting minimal impact on proteasome activity in normal cells. This selective action has driven the extensive development of copper complexes as anticancer agents targeting the proteasome [192,193]. Copper complexes have been reported to exert anticancer effects by targeting the ubiquitin–proteasome system (UPS) through five mechanisms: (1) reducing the thermal stability of ubiquitin [194]; (2) promoting the formation of spherical ubiquitin aggregates [195]; (3) inhibiting the activities of all three proteasome types [193]; (4) disrupting the channel gating of the 20S proteasome [196]; and (5) suppressing proteasome function via ROS generation and disassembly of the 26S proteasome [197].

In addition to inducing oxidative damage, accumulating evidence highlights the selective role of copper complexes as potential inhibitors of the UPS, contributing to their anticancer effects. First, the addition of Cu(II) to protein samples reduces the thermal stability of ubiquitin [194]. Second, incubation with Cu(II) promotes the formation of spherical ubiquitin aggregates, a process that can be blocked by Cu(II) chelation or reduction to Cu(I) [195]. Third, micromolar concentrations of Cu(II) inhibit the activities of all three proteasome types. Fourth, in cell-free systems, Cu(II) disrupts channel gating of the 20S proteasome without catalyzing redox reactions. Fifth, in HeLa cells, Cu(II) decreases proteasome activity via ROS-mediated inhibition and disassembly of the 26S proteasome [198]. Finally, several copper complexes demonstrate potent anticancer activity in vitro and in vivo by targeting the UPS.

The Cu(II)-bis(diethyldithiocarbamate) complex (CuET), identified by Bartek et al. as the active metabolite of DSF, preferentially accumulates in tumors and mediates its anticancer effects. Although CuET elicits phenotypes characteristic of proteasome inhibition, including ubiquitylated protein accumulation, it does not directly inhibit 20S or 26S proteasome activity. Rather, it interferes with the processing of ubiquitylated proteins by inhibiting the segregase activity of p97, specifically targeting its NPL4 subunit, which results in immobilization, functional disruption, and cell death [199]. CuET has demonstrated apoptotic activity in breast, prostate, and pancreatic cancer cell lines, providing a promising approach for treating p97-dependent malignancies.

Chen et al. evaluated the impact of disulfiram (DSF) and its copper conjugates on proteasome inhibition and apoptosis in chemoresistant MDA-MB-231 cells. Exposure to the Cu(II)-DSF complex triggered apoptotic hallmarks such as blebbing, chromatin condensation, and membrane disruption, ultimately causing cell death, whereas DSF alone showed no such effect [200,201].

Cu(II)-bis(8-hydroxyquinoline)complex (Cu(8 OHQ)_2_) has been identified as a potent, transient proteasome inhibitor capable of inducing apoptosis in human leukemia cells, with minimal impact on non-transformed, immortalized human cells under identical conditions. Experimental evidence suggests that the observed inhibition of chymotrypsin-like proteasome activity and apoptotic induction is not attributable to copper-mediated oxidative protein damage, but rather to the formation of a proteasome-inhibiting species within tumor cells [202].

5-chloro-7-iodo-8-hydroxyquinoline (CQ) has been shown to bind Cu(II) ions and form novel Cu(II) complexes that inhibit proteasome activity and induce apoptosis in cancer cells. CQ alone does not affect chymotrypsin-like proteasome activity. It has been proposed that, by targeting the elevated copper levels in cancer cells and tissues, treatment with compounds such as CQ may lead to the formation of tumor-specific proteasome inhibitors with therapeutic potential. In both normal and tumor tissues, copper predominantly exists in the Cu(I) state. Following CQ treatment, cellular copper interacts with CQ, converting Cu(I) to Cu(II), thereby significantly increasing Cu(II) levels in tumor tissues [203,204]. This Cu(I)-to-Cu(II) conversion in tumor cells is linked to CQ-induced proteasome inhibition.

Pyrrolidine dithiocarbamate (PDTC), the first compound in the dithiocarbamate family, has been shown to bind Cu(II), selectively inhibit the proteasome in cancer cells, and induce apoptosis in human breast and prostate cancer cells [205].

Cu(II)-Schiff base complexes have been shown to induce apoptosis in LNCaP prostate cancer cells by inhibiting chymotrypsin-like proteasome activity, independent of oxidative stress [206]. In particular, the Cu(II)-taurine Schiff base complex with 1,10-phenanthroline, [Cu(Tss)(phen)(H_2_O)] (Tss = taurine salicylic Schiff base), strongly inhibits proteasome activity and triggers apoptosis in MDA-MB-231 human breast cancer cells and Jurkat T leukemia cells [207].

Another study reported the synthesis of novel Cu(II)-amino acid Schiff base complexes: [Cu(Mvs)(phen), Cu(Vhn)(phen), Cu(Mvs)(Bpy), and Cu(Vhn)(Bpy)] (Mvs = L-methionine–o-vanillin Schiff base; Vhn = valine–2-hydroxy-1-naphthaldehyde Schiff base), incorporating either 1,10-phenanthroline (phen) or 2,2′-bipyridine (Bpy) as a secondary ligand. Cytotoxicity and antiproliferation assays against MDA-MB-231 and MCF-7 human breast cancer cells, as well as PC-3 prostate cancer cells, revealed varying effects. Notably, Cu(II) complexes containing phen as the second ligand effectively inhibited cell growth, suppressed proteasome activity, and induced cell death, highlighting the critical role of phen in determining cytotoxic activity [208].

Li et al. synthesized two Cu(II) complexes containing Schiff base and 1,10-phenanthroline, Cu(salicylamidate-4-fluorobenzoic acid)(phen) and Cu(salicylamidate-4-chloroanthranilic acid)(phen) and evaluated their antiproliferative effects against MDA-MB-231 cancer cells. Both complexes inhibited chymotrypsin-like proteasome activity, with the fluorinated complex showing greater potency. Treatment induced morphological changes and apoptosis, accompanied by PARP cleavage mediated via the proteasome target protein IkB-a. The complexes inhibited proteasomal chymotrypsin-like activity in a dose-dependent manner by targeting the catalytic β5 subunit of the 20S proteasome, as confirmed by molecular docking studies. Apoptotic induction was further supported by increased Bax expression, downregulation of Bcl-2 and caspase-3, and PARP cleavage [208,209].

The Cu(II)-pyrithione complex (CuPT) is known not only as an antifouling paint biocide but also for its potent anticancer activity [210]. CuPT suppresses cancer cell growth by targeting the active sites of 19S deubiquitinases (DUBs), UCHL5 and USP14, resulting in the accumulation of total and K48-linked ubiquitinated proteins, including CDKN1A, CDKN1B, BAX, and NFKBIA, as well as GFPu, a surrogate proteasome substrate. Consistently, CuPT inhibits 26S proteasome DUB activity in cell-free assays and competes with UbVS, a potent UCHL5 and USP14 inhibitor, for binding to these DUBs. While high doses of CuPT may cause off-target effects, low doses do not inhibit the chymotrypsin-like activity of the 20S proteasome. Its anticancer activity is mediated through apoptosis induction in multiple cancer cell lines (MCF-7, U266, and HepG2), primary monocytes from acute myeloid leukemia patients, and xenograft mouse models [211]. These findings highlight the broader role of metal-pyrithione complexes in targeting proteasomal DUBs [212,213].

The Cu(II)-hinokitiol complex (CuHK) has been identified as an inhibitor of DUBs but does not affect the chymotrypsin-like activity of the 20S proteasome. Inhibition of DUBs by CuHK leads to the accumulation of ubiquitinated proteins and GFPu in cancer cells (A549 and K562) and HEK293 cells, respectively. This, in turn, induces paraptosis-like cell death, a caspase-independent form of regulated cell death characterized by dilation of the ER and/or mitochondria. Notably, activation of ER stress through ATF4, rather than ROS generation, mediates CuHK-induced paraptosis in A549 and K562 cells. Whether CuHK-induced paraptosis involves activation of the ER-associated degradation (ERAD) machinery remains to be determined

Table 4 presents the copper complexes and their target as proteasome inhibitors.

An important challenge moving forward is to define the precise mechanisms and specific aspects of ubiquitin–proteasome system inhibition by copper complexes. Comprehensive profiling of their activity and potential adverse effects compared with other metal-containing agents, as well as distinguishing UPS-dependent from UPS-independent anticancer actions, will be essential for their development as clinically viable anticancer therapies.

## 6. Cuproptosis

The introduction of the concept of cuproptosis has sparked substantial interest and laid a theoretical foundation for the diagnostic and therapeutic use of copper in disease, particularly in oncology. Despite this progress, the clinical regulation of cuproptosis faces both opportunities and significant challenges. Research on copper chelators and carriers is still in its infancy, and disease-specific differences in copper metabolism complicate the development of copper-regulated drug design, chemotherapy, physical therapy, and immunotherapy, necessitating further mechanistic studies [215,216].

Copper is an essential trace element for biological organisms, and maintaining its homeostatic balance is critical for normal cellular function. Disruption of copper homeostasis leads to alterations in cellular structure and function. Excess intracellular copper induces oxidative stress and DNA damage, thereby triggering regulated cell death pathways such as apoptosis and necroptosis. In mitochondria, elevated copper levels can bind to lipoylated proteins of the tricarboxylic acid cycle, promoting their aggregation and causing proteotoxic stress, which ultimately activates a distinct form of regulated cell death known as cuproptosis. Consequently, targeting copper homeostasis has emerged as a promising strategy in oncology, involving either copper ion carriers to elevate intracellular copper and induce oxidative stress and cuproptosis, or copper chelators to reduce copper bioavailability [215,217].

The identification of cuproptosis by Tsvetkov et al. as a distinct copper-dependent form of cell death, independent of classical apoptotic or ferroptotic pathways, represents a major advance in our understanding of copper-mediated cytotoxicity [27].

Cuproptosis is triggered by elevated intracellular copper ions. At present, two main therapeutic strategies have been explored for inducing copper-dependent cell death. One approach involves the use of copper ion chelators or copper-complexing agents, which reduce intracellular copper levels through direct binding. In this context, Cu(I) preferentially coordinates with diphosphine, nitrogen, and polypyridine ligands, whereas Cu(II) binds to Schiff bases, DSF, and p-chlorobenzoic acid, collectively inhibiting tumor cell proliferation and metastasis. The second strategy employs copper ion carriers, which transport copper into cells, increasing intracellular copper concentrations and inducing tumor cell death through ROS generation or proteasome inhibition, thereby exerting antitumor effects [214,215,216,217].

Elucidating the mechanisms underlying cuproptosis provides a promising framework for drug discovery, particularly for the development of copper ionophores and other cuproptosis-associated agents with potential applications in future cancer therapies. Copper ionophores—lipid-soluble molecules that reversibly bind copper ions—played a central role in the discovery of cuproptosis and may significantly influence antitumor treatment strategies [218]. These compounds facilitate copper transport across cellular and mitochondrial membranes. Notably, agents such as Elesclomol (ELC) and Disulfiram (DSF) promote cell death by delivering copper into cells and mitochondria, resulting in DLAT oligomerization, destabilization of iron–sulfur (Fe–S) clusters, and Npl4 interaction [219].

**Figure 5:**
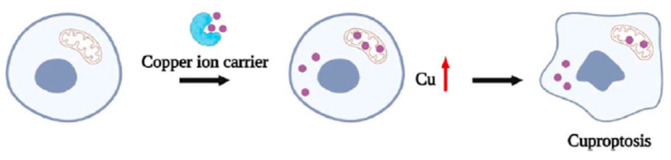
Copper ionophores in cuproptosis (adapted from [218]).

In contrast to metal chelators, which reduce metal ion concentrations in the body, ionophores promote the intracellular accumulation of metal ions. An effective copper ionophore must exhibit thermodynamic stability combined with kinetic lability and possess a moderate affinity for copper, enabling efficient metal release at the target site. Ionophores with weak copper binding fail to transport the metal effectively, whereas those with excessively high affinity retain copper and prevent its release. Thus, an optimal ionophore coordinates copper in regions of high concentration and releases it where copper levels are lower. Copper ionophores bind extracellular copper to form complexes that traverse the lipid bilayer via passive diffusion (Figure 5), making lipophilicity or hydrophobicity a key determinant of membrane permeability [215,216,217,218].

Copper ionophores can be broadly classified into four major categories: dithiocarbamates (DTCs), thiosemicarbazones (TSCs), hydroxyquinolines (HQs), and hydroxyflavones (HFs) [219,220,221].

Among the various copper ionophores identified to date, DSF and ELC are the most extensively studied. DSF, the oxidized form of diethyldithiocarbamate, belongs to the DTC class and has recently gained attention for its ability to promote metal accumulation in cancer cells and induce regulated cell death. Preclinical studies have demonstrated that DSF inhibits tumor growth and that co-delivery of DSF and Cu(II) markedly enhances intracellular ROS generation. The Cu–DSF complex downregulates XIAP and suppresses NF-κB signaling through excessive ROS production. In TRAMP-C1 cells, this complex releases Cu(II), further increasing ROS levels while depleting glutathione (GSH), the primary cellular antioxidant, thereby rendering cells more susceptible to oxidative damage [222,223].

ELC is another well-characterized copper ionophore that forms a stable complex with Cu(II) and facilitates its transport into mitochondria, where it induces oxidative stress. Treatment of cells with the Cu–ELC complex results in marked inhibition of cell proliferation. In K562 cells, this complex also induces DNA double-strand cleavage. Mechanistically, elesclomol delivers copper into cells, where Cu(II) is subsequently reduced to Cu(I). The reduced Cu(I) then participates in Fenton-like reactions, generating superoxide (O_2_^−^) and ultimately hydrogen peroxide (H_2_O_2_), thereby promoting oxidative damage [224].

Mediator of ErbB2-driven cell motility (Memo), a copper-dependent oxidoreductase, promotes ROS production within cellular structures associated with migration and is essential for breast cancer cell migration and invasion in vitro, as well as for spontaneous lung metastasis in breast cancer xenograft models in vivo. Treatment with the copper chelator tetrathiomolybdate (TTM) markedly reduces intracellular copper levels, downregulates Memo expression, impairs tumor angiogenesis, suppresses tumor growth, and significantly diminishes the invasive potential of breast cancer cells [212]. TTM is an oral copper chelator that can suppress tumor growth and angiogenesis, as well as function as a cuproptosis inhibitor. It primarily forms high-affinity complexes with copper, chelates copper in the bloodstream, inhibits NF-κB signaling, and decreases the production of angiogenic factors. Additionally, TTM suppresses the activity of LOX and focal adhesion kinase, lowers matrix metalloproteinase levels, markedly reduces cancer cell motility and invasiveness, promotes tumor cell apoptosis, and impedes metastasis in head and neck cancers. Clinical studies have also demonstrated TTM’s anti-angiogenic effects in patients with malignant pleural mesothelioma [213,225].

Although preclinical studies have shown that copper chelation or induction of copper ion-dependent cell death via copper ionophores can exert promising anticancer effects, several challenges hinder their clinical translation, including limited specificity and short half-life. Developing new strategies is crucial to overcome these barriers and enable effective cancer therapies targeting copper-dependent mechanisms. Such strategies may involve selectively increasing copper ion levels in cancer cells, protecting healthy cells from copper-induced damage, and prolonging the duration of cuproptosis [226,227].

Since cuproptosis is driven by intracellular copper metabolism, it can induce cytotoxicity in both cancerous and normal cells, making careful regulation of intracellular copper levels essential. Nanomedicine has emerged as a promising strategy to address this challenge, as it can improve drug solubility, extend circulation time, enable targeted delivery, and reduce off-target side effects [228,229]. As a result, the recent understanding of cuproptosis has spurred considerable advances in the development of nanomaterials, showing promising potential for cancer therapy. For instance, copper and copper oxide nanoparticles have attracted significant interest in cancer research, offering multiple benefits such as improved drug stability, optimized biodistribution, enhanced therapeutic index, and targeted delivery of active agents to specific sites via both active and passive targeting strategies [230,231]. Copper and copper oxide nanoparticles can supply copper ions to cells, but these ions may also trigger a Fenton reaction with hydrogen peroxide, producing cytotoxic hydroxyl radicals. Additionally, the intracellular conversion of Cu(II) to Cu(I) in target cancer cells must be taken into account for effective cuproptosis, which relies on DLAT aggregation and Fe-S cluster loss caused by excessive Cu(I). Therefore, careful nanomedicine design and dose optimization are essential to induce efficient cuproptosis. Finally, combining cuproptosis-inducing strategies with other therapeutic approaches that exploit different cell death mechanisms may further enhance anticancer efficacy [221,232]. Moreover, the rapid advancement of nanotechnology in medicine has driven the development of novel copper-based nanomaterial complexes. However, studying these materials remains a significant challenge in the field. Further research is required to address issues such as avoiding autoimmune responses, minimizing side effects, enhancing drug targeting and stability, and other related concerns. Current evidence indicates that achieving these goals and successfully integrating cuproptosis-based therapies into clinical cancer care remains a formidable challenge.

## 7. Copper Complexes in Clinical Trials

Copper-based complexes that strongly inhibit cancer cell proliferation in animal models highlight the significance of copper compounds in medicinal chemistry and are regarded as one of the most promising classes of anticancer agents. Mixed chelate copper complexes, including drugs like Cassiopeias and Elesclomol (ELC), have also been evaluated in clinical trials.

**Figure 6:**
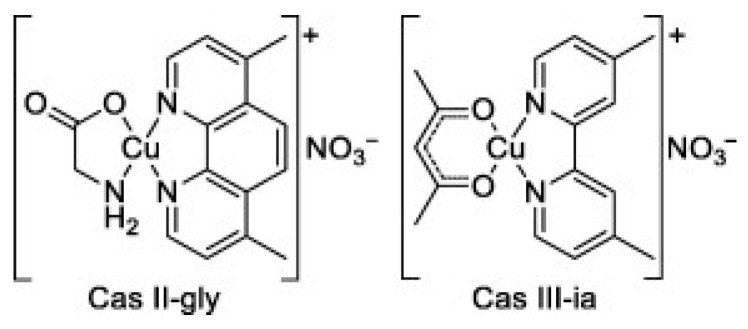
Chemical structures of Casiopeina II-gly and Casiopeina-III-ia.

Casiopeínas are a family of copper coordination compounds that have shown promising potential in the treatment of colorectal cancer and acute myeloid leukemia. Several Casiopeínas compounds have demonstrated significant therapeutic efficacy, with Casiopeina III-ia and Casiopeina II Gly (Figure 6) advancing to multiple clinical trials as treatments for leukemia [233].

Several mechanisms have been proposed for the action of Casiopeínas, including ROS generation, phosphate hydrolysis, DNA damage, and DNA intercalation [234].

Casiopeina IIIia is presently undergoing phase I clinical trials, and studies suggest that its anticancer activity involves DNA fragmentation via intercalation, mediated by copper reduction and the production of ROS. Casiopeina IIgly induces cell death by triggering oxidative damage, which results in mitochondrial dysfunction and ultimately apoptosis via both caspase-dependent and caspase-independent pathways. Three key effects of Casiopeina treatment have been identified: (a) disruption of signaling pathways involved in apoptosis, (b) interference with metabolic pathways, and (c) activation of immune responses [235,236].

**Figure 7:**
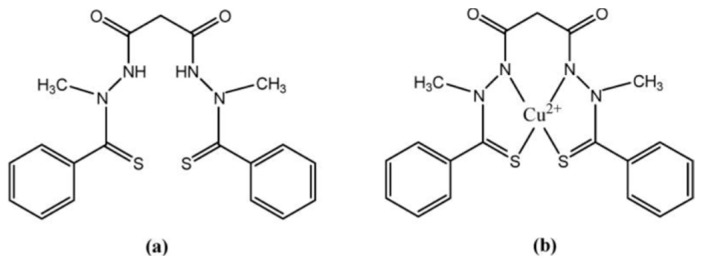
Chemical structure of Elesclomol (**a**) and Complex Cu(II)-Elesclomol (**b**).

ELC is a mitochondria-targeting copper ionophore developed for chemotherapy. This bis(thiohydrazide) amide forms a 1:1 complex with copper(II) in the extracellular space, creating a membrane-permeable species capable of delivering copper directly to mitochondria (Figure 7). Following the reduction of copper(II), copper(I) is released within the mitochondria [13]. Inside mitochondria, the ELC–Cu(II) complex is reduced to Cu(I), initiating the production of ROS. ELC induces oxidative stress in cancer cells by elevating ROS levels beyond tolerable thresholds, achieved both through increased ROS generation and the activation of a transcriptional program characteristic of an oxidative stress response. Previous studies have shown that ELC can increase ROS production and induce a dose-dependent inhibition of mitochondrial NADH–ubiquinone oxidoreductase activity. The elevated ROS levels and associated cytotoxicity caused by ELC may result from mitochondrial oxidative phosphorylation uncoupling and/or inhibition of electron transport [66]. Clinical trials have identified ELC-sensitive cancers, including lung cancer, breast cancer, melanoma, and ovarian cancer [237,238,239].

The primary clinical applications of ELC include: (I) acting as a ‘Trojan-horse’ to deliver copper in cases of copper deficiency, (II) demonstrating effectiveness against various multi-drug-resistant cancers, and (III) participating in pathways associated with ROS generation. Although ELC has shown significant therapeutic potential, its efficacy as a single agent can be influenced by multiple factors. Therefore, combining ELC with other therapeutic agents represents a strategy to enhance its anticancer activity. Overall, due to its selectivity and central mechanisms of action, ELC is considered a promising anticancer agent [237,238].

**Figure 8:**
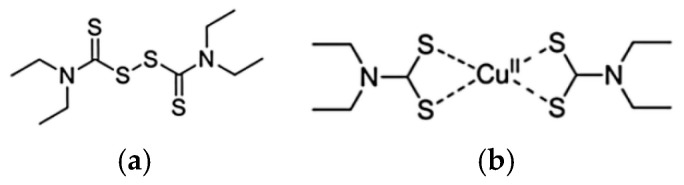
Chemical structure of Disulfiram (**a**) and DDTC-Cu(II) (**b**).

Preclinical evidence indicates that DSF exhibits anticancer activity, which is markedly enhanced in the presence of Cu(II) ions. DSF is metabolized to diethyldithiocarbamate (DDTC), a strong metal ion chelator that forms DDTC–metal complexes upon chelation (Figure 8).

It has been proposed that the accumulation of DDTC–copper complexes, together with depletion of bioavailable copper, is detrimental to cancer cell viability [239,240]. Elevated serum levels of ceruloplasmin, the primary copper-transport protein, are linked to angiogenesis and have been reported in multiple cancer types. Furthermore, depletion of copper has been shown to suppress several angiogenic factors, including VEGF, IL-1, and IL-8 [155]. Recent studies demonstrate that the combination of disulfiram and copper (DSF/Cu) effectively targets proteasome function, aldehyde dehydrogenase (ALDH) activity, and NF-κB signaling, thereby increasing the cytotoxic efficacy of multiple chemotherapeutic agents and helping to overcome drug resistance [240,241]. Another proposed mechanism underlying cancer cell death is the induction of oxidative stress, which can result in cellular damage and apoptosis. DSF-induced generation of ROS has been shown to activate the proapoptotic ROS–JNK signaling pathway [157] and to initiate the UPR, leading to autophagy-dependent apoptosis in cancer cells [242].

Numerous phase I–III clinical trials are currently evaluating the DSF/Cu(II) combination for cancer therapy. These studies encompass a wide range of malignancies, including breast, prostate, pancreatic, liver, and non-small cell lung cancers, as well as testicular germ cell tumors, multiple myeloma, sarcoma, melanoma, and glioblastoma. In light of in vitro evidence demonstrating that DSF’s anticancer activity is dependent on copper ions, many trials are assessing DSF combined with copper either alone or alongside standard chemotherapeutic regimens in refractory disease. Although clinical outcomes to date have been modest, there remains optimism that DSF may achieve therapeutic benefit when administered with copper or as an adjunct to established treatments. Furthermore, the development of novel DSF/Cu(II) formulations employing nanotechnology-based drug delivery systems is expected to play a crucial role in advancing the clinical translation of DSF for cancer therapy [243,244].

## 8. Conclusions

Research over recent decades has demonstrated the promising potential of copper complexes in cancer therapy. The integration of the aforementioned strategies with rational ligand design enables exploitation of the unique properties of Cu(II) complexes, providing a foundation for the development of more effective and targeted anticancer treatments. Moreover, the versatility of Cu(II) complexes in integrating multiple mechanisms of action with advanced therapeutic approaches presents new opportunities to overcome drug resistance, enhance cancer cell specificity, and ultimately minimize damage to healthy tissues. As efforts to develop next-generation Cu(II)-based therapeutics advance, combining coordination chemistry with modern biomedical technologies offers significant potential for more precise and personalized cancer treatments. Preclinical studies have demonstrated the anticancer efficacy of copper complexes across various cancer types. While the precise molecular targets and mechanisms of action remain incompletely understood, several potential mechanisms have been proposed and are discussed in this article. Evidence suggests that copper complexes act through distinct mechanisms and demonstrate promising in vitro and in vivo anticancer activity, positioning them as potential alternatives to traditional platinum-based drugs. Additionally, copper complexes display a broader spectrum of activity and reduced toxicity, offering the potential to overcome limitations associated with platinum therapies.

## Figures and Tables

**Table 1:** Evaluation of DNA-binding studies for various Cu(II) complexes.

Copper-Complex	Type of Interaction	Biological Effect/Antitumor Activity	Reference
Cu-2-(5-(triflorometil)-2-metoxifenilimino) metil)-4,6-dichlorofenol	Intercalation	HeLa cervical carcinoma line A549 lung cancer cells	[75]
Cu-2-(4-sulfametazin)hidrazono-5,5- dimetilchlohexan-1,3-dione	Electrostatic interaction/ Groove binding	Antioxidant effects	[76]
Cu-1-methyl-l-tryptophan	Intercalation/ electrostatic interaction	Affinity for DNA/ Potential antitumor agents	[77]
Cu-Schiff bases of 2-hydroxy-1-naphthaldehyde Cu-Schiff bases of 4-amino-acetophenone	Groove binding	Potential antitumor agents	[78]
Cu-Esculetin	Minor groove binding	Strong photodynamic therapy potential	[79]
Cu-((2-(pyridin-2-yl)-1H-benzo[d]imidazol- 1-yl)methyl)quinolone	Intercalation	Inducing cell apoptosis	[80]
Cu-5-methyl-2-phenyl-1,2-dihydro-3H-pyrazole-3-one Cu-3-methyl-1-phenyl4-[(E)- phenyldiazenyl]−4,5-dihydro-1H-pyrazole-5-ol	Intercalation	Interaction with DNA/Potential antitumor agents	[81]
Cu-benzimidazole derivatives	Intercalation/ Groove binding	MDA-MB 231 breast cancer cells	[82]
Cu-polypyridyl	Intercalation/ Groove binding	MCF-7 breast cancer cells line	[83]
Cu-tritriazole	Groove binding	Breast cancer cells lines	[84]
Cu-vanillin Schiff base—naproxen	Groove binding	Breast cancer cells lines	[85]
Cu-thioflavin-T-based derivative 4′-bis(pyridine-2- ylmethyl)amiono-2-phenylbenzothiazole)	Intercalation	A549 lung cancer cells MCF-7 breast cancer cells	[86]
Cu-N-subtituted sulphonamides	Groove binding	HeLa cervical carcinoma line DLD-1 colorectal carcinoma line	[21,22]

**Table 2:** Cu(II) complexes as DNA photocleavage agents.

Copper-Complex	Biological Effect/ Antitumor Activity	Reference
Cu(II)-ferrocenyl-L-amino acid	HeLa MCF-7 cancer cells	[120]
Cu(II)-2-[(pyridin-2-yl)methyleneamino] phenol-imidazole	HeLa cells	[121]
Cu(II)-*N*,*N*,*N*-donor dipicolylamine	HeLa MCF-7 cancer cells	[122,123]
Cu(II)-*N*,*N*,*O*-tridentate Schiff-base derivatives	SCC15 (human squamous cell carcinoma) BCC (basal cell carcinoma)	[110]
Cu(II)-L–lysine and L-arginine appended to an anthracene unit and phenanthroline bases	A549 (human lung carcinoma) HaCaT (human epidermal keratinocytes) MDA-MB-231(breast cancer cells)	[124]
Cu(II)-Schiff Base	SCC15 BCC	[125]

**Table 3:** Copper complexes as topoisomerases inhibitors.

Copper Complexes as Top1 Inhibitors	Copper Complexes as Top2 Inhibitors
Cu(II)-oxindolimine [141]	Cu(II)-α-(N)-heterocyclic thiosemicarbazone [146]
Cu(II)-hydrazone [147]	Cu(II)-pyridine-thiosemicarbazone [148]
Cu(II)-plubagin [149]	Cu(II)-piperazine-thiosemicarbazone [150]
Cu(II)-phenanthroline-aminoacide [151]	Cu(II)-thiazole-thiosemicarbazone [145,152]
Cu(II)-phenantroline-pyrophosphate-bridge [153]	Cu(II)-proline-thiosemicarbazone [154]
Cu(II)-Sn_2_(IV)-phenantroline and ethylenediamine [155]	Cu(II)-quinoline-thiosemicarbazone [156]
Cu(II)-Schiff base, Cu(II)-Sn(IV)-Schiff base [157,158]	Cu(II)-naphthoquinone-thiosemicarbazone [159]
Cu(II)-chalcone derived thiosemicarbazone [160]	Cu(II)-carbohidrazone [161]
Cu(II)-tetrazolo[1,5-a]pyrimidine [162]	Cu(II)-chromone [163]
Cu(II)-dipeptide piperazine-bridged [164]	Cu(II)-quinolinone [165]
Cu(II)-Elesclomol [166]	Copper complex as Top1/Top2α dual inhibitor
Cu(II)-S-benzyldithiocarbazate and 3-acetylcoumarin [167,168]	Cu(I)-bis-pyrazolyl carboxylate-phosphine [169]

**Table 4:** Copper complexes as proteasome inhibitors.

Copper Complexes	Target	References
CuET	20S proteasome NPLOC4	[199]
CuDSF	20S proteasome	[200,201]
Cu(8-OHQ)_2_	20S proteasome	[202]
CuCQ	20S proteasome	[204]
CuPDTC	20S proteasome	[205]
Cu-Schiff base-phen	20S proteasome	[206,207]
CuPT	UCHL5 USP14	[210]
CuHK	UCHL5 USP14	[214]

## Data Availability

No new data were created or analyzed in this study. Data sharing is not applicable to this article.
